# The relationship between inflammatory dietary pattern in childhood and depression in early adulthood

**DOI:** 10.1016/j.bbih.2019.100017

**Published:** 2019-12-15

**Authors:** Xiao Cong, Melissa Tracy, Lynn S. Edmunds, Akiko S. Hosler, Allison A. Appleton

**Affiliations:** a Department of Epidemiology and Biostatistics, School of Public Health, State University of New York at Albany, 1 University Place, Rensselaer, NY 12144, United States; b Division of Nutrition, New York State Department of Health, USA

**Keywords:** ALSPAC, Cohort, Children, Depression, Inflammatory dietary pattern, Inflammatory biomarkers, C-reactive protein, Interleukin-6, Reduced rank regression

## Abstract

**Background:**

Inflammation may be a hidden process in the relationship between dietary intake and depression, but no study has evaluated the role of diet and inflammation jointly in explaining depression risk in early life. The current study aims to investigate the relationship between inflammatory dietary pattern (IDP) in childhood and depression in early adulthood.

**Methods:**

This study used data prospectively collected over 10 years from the Avon Longitudinal Study of Parents and Children (ALSPAC) cohort (n ​= ​6939) free from depression at baseline (age 8.5 years). An IDP score was empirically derived via reduced rank regression and stepwise linear regression based on dietary intake data from the food frequency questionnaire at 8.5 years and levels of inflammatory biomarkers, C-reactive protein and interleukin-6, at 9.5 years. At age 18 years, depression cases were identified via the International Statistical Classification of Diseases, 10th Revision (ICD-10) diagnosis and the Clinical Interview Schedule-Revised (CIS-R) depression score. Logistic regression models were constructed to examine the relationship between the IDP score and risk of depression adjusted for potential confounders. Analyses stratified by weight status were also conducted. Multiple imputations were utilized to minimize bias due to loss-to-follow-up.

**Results:**

Participants in the highest tertile of IDP score had 1.34 times odds to develop depression compared to those in the lowest tertile (95% CI, 1.08–1.66; *P*-trend<0.01), after dietary misreporting status and energy intake were adjusted. After all covariates were adjusted, the relationship between IDP tertiles and depression was attenuated (highest tertile vs. lowest tertile: OR ​= ​1.21; 95% CI, 0.96–1.51); in addition, the relationship was marginally significant among participants who were not overweight or obese (p ​< ​0.10) but not significant among participants who were overweight or obese.

**Conclusions:**

Higher IDP in childhood seems to be associated with higher depression risk in early adulthood. The study provides preliminary evidence that chronic inflammation may underlie the relationship between diet and depression even for children, especially those who are not overweight or obese.

## Highlights

• Inflammatory diet in childhood increases depression risk in early adulthood.
• The relationship is more robust among children who are not overweight or obese.
• Inflammation may underlie the link between diet and depression even for children.
• Anti-inflammatory diet could be recommended for children to prevent depression.

## 1 Introduction

Depression influences about 16% of Americans over their lifetime, with a quarter of these cases starting before age 20 years (Kessler et al., 2003; Kessler et al., 2005; Murray and Lopez, 2013). Depression occurring in adolescence and early adulthood tends to persist into later life stages and can profoundly affect academic performance, social connections, and physical and mental health later in life, causing large costs to both individuals and society (Fergusson and Woodward, 2002; Fombonne et al., 2001). Childhood is a sensitive period of human development when adverse exposures can influence mental and physical disease risk for a lifetime (Ben-Shlomo and Kuh, 2002). Therefore, identifying early life etiologic factors that are modifiable may be particularly useful in reducing the burden of depression in the population. Where much depression research focuses on the psychosocial determinants of the disease (Bowes et al., 2015; Fletcher, 2009), researchers are increasingly considering whether dietary factors also play a role, though few have considered diet in early life as contributing to later life depression risk.

Recent prospective studies among adults have indicated that healthy dietary patterns (featuring vegetables, fruits, whole grains, fish, and mushrooms), such as the Mediterranean style diet (Rienks et al., 2013) and the Prudent diet (Jacka et al., 2014; Ruusunen et al., 2014), decrease depression risk, while unhealthy dietary patterns (featuring red meat, fried foods, refined grains, highly processed foods, and sugar sweetened beverages), such as the Western diet (Jacka et al., 2014; Le Port et al., 2012), increase depression risk (Jacka et al., 2014; Le Port et al., 2012; Rienks et al., 2013; Ruusunen et al., 2014). For example, one study of 6060 women aged 50–55 years found that higher adherence to the Mediterranean style diet at baseline was associated with lower incidence of depressive symptoms 3 years later (Rienks et al., 2013). Moreover, while it has been shown that dietary habits formed in childhood tend to be maintained into adulthood (Mikkila et al., 2005), knowledge of the effects of diet early in life on mental health is limited (Khalid et al., 2016; O’Neil et al., 2014). One cross-sectional study among 5003 Chinese adolescents aged 11–16 years derived three dietary patterns via factor analysis and indicated that the “snack” and “animal food” patterns were associated with higher levels of depression, while the “traditional” pattern (fresh fruits and vegetables, soy products, gruel, whole grains, etc.) was associated with lower levels of depression (Weng et al., 2012). Another cross-sectional study of 1799 adolescents found higher adherence to the “Western” dietary pattern and less adherence to the “Healthy” dietary pattern were significantly associated with attention deficit hyperactivity disorder (ADHD) diagnosis (Howard et al., 2011). While these studies and similar work are suggestive of an association between childhood dietary pattern and risk of mental health and behavioral problems later in life, studies in this area are subject to some limitations, including an overreliance on cross-sectional designs which raise concerns about reverse causality (Oddy et al., 2009; Oellingrath et al., 2013; Weng et al., 2012), short follow up period between exposure and outcome assessments (Jacka et al., 2013; McMartin et al., 2012; Trapp et al., 2016), and emphasis on childhood externalizing behavior problems, such as ADHD (Azadbakht and Esmaillzadeh, 2012; Howard et al., 2011; Khalid et al., 2016; O’Neil et al., 2014; van Egmond-Froehlich et al., 2012). Therefore, research is needed to better characterize the long-term prospective association between childhood dietary practices and depression risk later in life.

A plausible biologic mechanism linking early life dietary practices with depression risk is systemic inflammation. As part of the adaptive reaction to infection from the immune system, inflammation measured by levels of serum inflammatory markers, such as interleukin 6 (IL-6) and C-reactive protein (CRP), can trigger a set of metabolic syndromes and behavioral regulations (Yaffe et al., 2004). A large body of work has shown the bidirectional relationship between inflammation and depression. In particular, while some studies show that individuals with depression have a higher risk of developing inflammation (Copeland et al., 2012; Deverts et al., 2010; Duivis et al., 2011; Stewart et al., 2009), others show that inflammation can contribute to depression (Gimeno et al., 2009; Khandaker et al., 2014; Valkanova et al., 2013). For example, one prospective study among 4415 youths (the dataset used for the current analysis) has indicated that higher IL-6 levels at age 9 years were associated with an increased risk of depression at age 18 years (Khandaker et al., 2014). Other research suggests that an unhealthy diet that is high in saturated fats, trans fatty acids, sugar, and refined starches, but low in omega-3 (n-3) unsaturated fats (e.g. from fish oil), whole grains, fibers (from fruits and vegetables), and natural antioxidants may stimulate inflammation (Kaplan et al., 2015; Loprinzi et al., 2015). Taken together, these studies suggest that chronic inflammation may be a process through which dietary intake influences depression (Kiecolt-Glaser, 2010; Kiecolt-Glaser et al., 2015).

Compared to individual food items, dietary patterns, measured *a priori* via dietary indices (e.g. Healthy Eating Index) or derived *a posteriori* via statistical methods (e.g. factor analysis), can incorporate complex interactions among foods/nutrients and can better predict disease risk (Hu, 2002). A few studies among adults have integrated information on diet and inflammation to study the joint contribution of these factors on depression risk. Four studies utilized an *a priori* Dietary Inflammatory Index (DII) (Shivappa et al., 2014) and found that higher DII scores were associated with higher depression risk (Adjibade et al., 2017; Akbaraly et al., 2016; Sanchez-Villegas et al., 2015; Shivappa et al., 2016). However, like other *a priori* dietary indices, the DII focused only on selected aspects of diet and did not use all of the available dietary intake information from the study samples (Hoffmann et al., 2004; Hu, 2002). To overcome limitations of the *a priori* dietary index, one study empirically derived an Inflammatory Dietary Pattern (IDP), which incorporated biomarkers of inflammation and self-reported information on dietary intake via reduced rank regression (RRR) (Lucas et al., 2014). Through RRR, dietary patterns that maximally explain variation in inflammatory markers are empirically derived and may better predict disease, since both empiric information from study data and theorized information about disease-related variables are utilized (Hoffmann et al., 2004; Lucas et al., 2014). Using this approach, the study examined 43,685 Nurses’ Health Study participants aged 50–77 years and found those in the top IDP quintile exhibited 40% higher odds of developing depression compared with women in the lowest quintile, after confounders were controlled, over 12 years of follow up (Lucas et al., 2014). Taken together, these studies indicate that an inflammatory diet may contribute to depression risk and that empirically derived biomarker-based IDP measures may be more valid and precise than *a priori* measures. However, this approach has not been applied to a younger population, and thus it is not clear if an inflammatory dietary pattern in childhood could likewise be derived to predict depression risk. Furthermore, compared with traditional mediation analysis that investigates the mediating effect from inflammation on the relationship between diet and depression, RRR can identify the components of the diet that are most relevant to inflammation.

In addition, adipose tissue is a rich source of inflammatory factors and the relationship between adiposity and depression is bidirectional (Shelton and Miller, 2011). Adipose tissue produces inflammatory factors, including cytokines such as IL-6, TNF-α, and chemokines, which in turn may activate widespread immune reaction, potentially leading to or exacerbating inflammation-related diseases, including depression (Shelton and Miller, 2011). Previous studies that investigated the association between inflammatory diet and depression among adults indicate there may be a modifying effect of weight status. For example, a prospective study among 15,093 adults found a stronger association between DII score and depression among obese vs. non-obese participants (Sanchez-Villegas et al., 2015). It is not known whether such weight-status patterning in the inflammatory diet and depression association would be similarly evident earlier in the life course.

In the current study, we integrated biomarkers of inflammation with a widely-used dietary assessment to examine the association between inflammatory dietary patterns in childhood and incident depression in young adulthood. We leveraged nearly 10 years of prospectively collected life course information from the Avon Longitudinal Study of Parents and Children (ALSPAC), a large population-based birth cohort study from the United Kingdom. We hypothesized that children with higher IDP scores would have higher risk of depression in young adulthood compared to children with lower IDP scores, controlling for relevant social, behavioral, and demographic confounders. We also considered whether the association was modified by weight status. To our knowledge, this study is among the first to consider an inflammatory dietary pattern in childhood in association with depression risk later in life.

## 2 Material and methods

### 2.1 Study sample

The study sample originates from ALSPAC, an ongoing population-based trans-generational prospective study. The core aim of ALSPAC is to examine the effects of a wide range of social, biological, and environmental factors on women’s pregnancies and their children’s physical and mental health. Pregnant women who lived in Avon County in Southwest England and had an estimated delivery date between April 1st, 1991 and December 31st, 1992 were eligible to participate. About 85% of all eligible women enrolled, forming a cohort of 14,541 pregnant women who gave birth to 13,988 children alive at 1 year of age (phase I recruitment). 713 children were additionally recruited when they were around 7 years old (phase II and phase III recruitment) (Boyd et al., 2013; Fraser et al., 2013; Golding et al., 2001). The study website contains details of all the data that are available through a fully searchable data dictionary and variable search tool (http://www.bristol.ac.uk/alspac/researchers/our-data/). Questionnaires were mailed to mothers four times during pregnancy. After the children were born, child-related questionnaires were sent to mothers twice a year and mother/partner-related questionnaires were sent once a year. The survey collected data ranging from socio-demographic characteristics to life style factors, physical health, and mental health for study children, mothers, and partners. Researchers also collected biological and genetic samples (e.g. blood, urine, hair, nail, etc.) from both mothers and children during their clinic visits at certain time points (Boyd et al., 2013; Fraser et al., 2013; Golding et al., 2001; Ness, 2004).

**Fig. 1:**
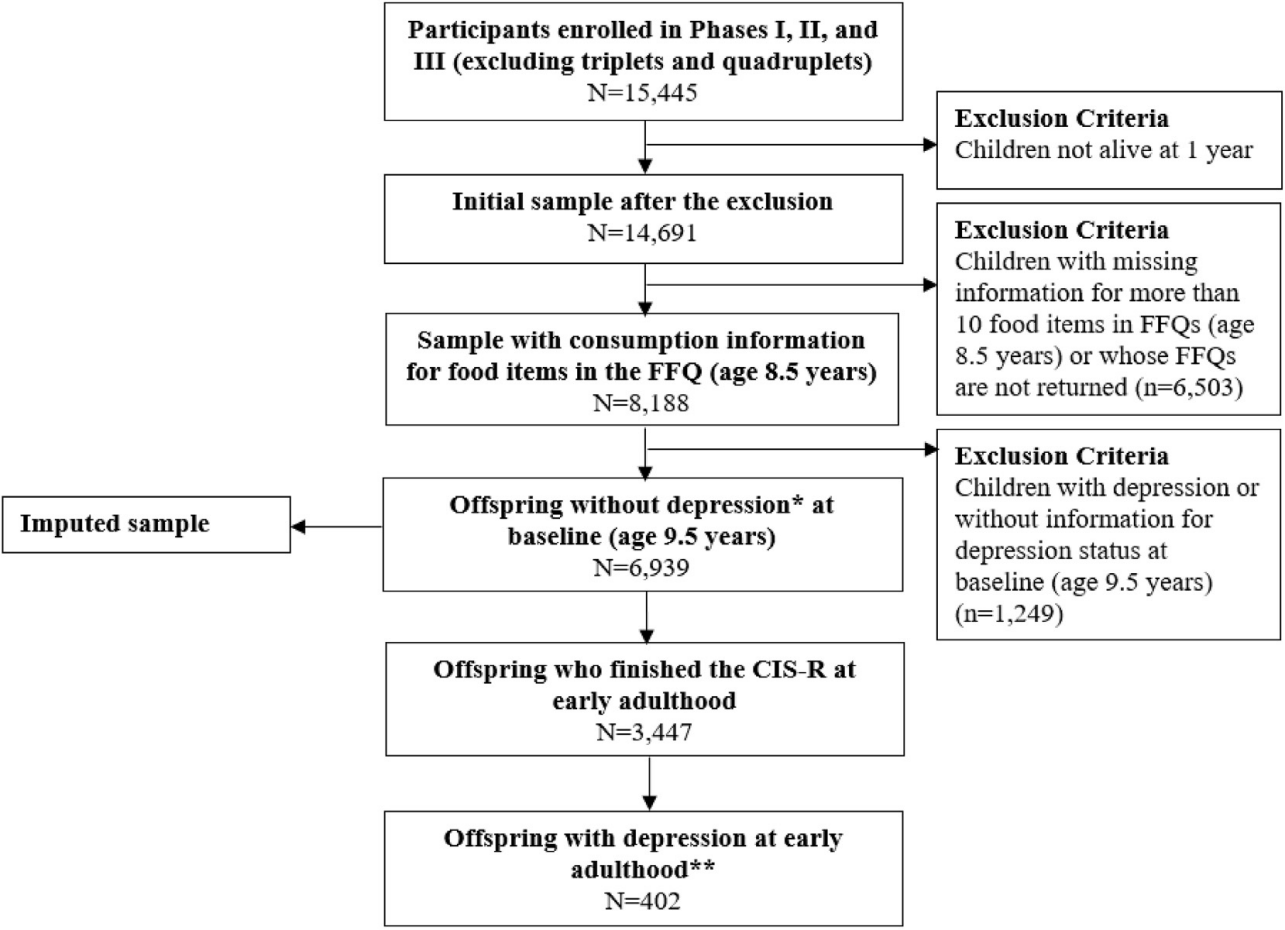
Flowchart of the study sample in the ALSPAC. ALSPAC, Avon Longitudinal Study of Parents and Children; FFQ, Food Frequency Questionnaire; CIS-R, Computerized Interview Schedule-Revised; SMFQ, Short Moods and Feelings Questionnaire; ICD-10, the International Statistical Classification of Diseases, 10th Revision. * Baseline depression (at age 9.5 years) was assessed via the SMFQ reported by mothers/main caregivers. ** Participants were defined as depression cases if they had depression score ​≥ ​9 (out of 21) measured via the CIS-R or had ICD-10 diagnosis of depression at early adulthood (i.e., at age 18 years).

The present study uses data from a subset of ALSPAC participants, specifically children who had available dietary intake information at age 8.5 years. To ensure incident cases of depression were identified at follow up, we excluded children with depression at age 9.5 years (based on a score of ≥12 on the Short Moods and Feelings Questionnaire) (Angold et al., 1995). Thus, the base analytic sample included 6939 participants (Fig. 1). The ALSPAC Law and Ethics Committee, the ALSPAC Local Research Ethics Committee, and the Institutional Review Board of the University at Albany, State University of New York authorized the study protocol.

### 2.2 Depression in early adulthood

During the clinic visit when participants were about 18 years old, 3447 (49.7% of the total sample of 6939) completed the computerized version of the Clinical Interview Schedule-Revised (CIS-R), a standardized and validated tool widely utilized to measure depression and anxiety (Lewis et al., 1992). The CIS-R incorporates core symptoms of depression from *International Statistical Classification of Diseases,* 10th *Revision* (ICD-10) to produce a composite depression score (ranging from 0 to 21) that includes sub-scores from five sources---depression, fatigue, concentration, sleep issues, and depressive thoughts---to indicate severity of depressive symptoms in the previous week (Bowes et al., 2015; Khandaker et al., 2014). We combined the criteria (score 9 as cut-point of the CIS-R depression score) in a previous study (Khandaker et al., 2014) with the ICD-10 diagnosis of probable depression to more comprehensively identify probable depression cases in early adulthood. Among the 3447 participants, 402 (11.7%) were classified as depression cases (Fig. 1).

### 2.3 Inflammatory dietary pattern score in childhood

The IDP score at age 9 years was derived by synthesizing parent-reported information on diet from a Food Frequency Questionnaire (FFQ) and child biomarkers of CRP and IL-6, and by conducting the RRR analysis. These variables and derivations are described in turn below.

**Diet.** When children were 8.5 years old, dietary intake was evaluated via a FFQ completed by mothers, or main caregivers (University of Bristol, 2000). The FFQ contains a series of questions inquiring about the “current” consumption frequency of a wide range of food and drinks, including 90 items (87 detailed items and 3 general items [“milk”, “bread”, and “spread”]). Participants were excluded if they failed to return the FFQ or had more than 10 food items missing over the 90 items in the FFQ. If ten or fewer items were unanswered, FFQ data were utilized and the missing items were assigned a value of 0 (Emmett et al., 2015; Northstone, Joinson, Emmett, Ness and Paus, 2012).

Most items had standardized options reflecting the consumption frequency of each food item: (1) “never or rarely”; (2) “once in 2 weeks”; (3) “1–3 times per week”; (4) “4–7 times per week”; or (5) “more than once per day”. To facilitate quantitative analysis, data were converted into a weekly basis (i.e. 0; 0.5; 2; 5.5; and 10 times per week corresponding to the five frequency options) (Emmett et al., 2015; Northstone and Emmett, 2005; Northstone et al., 2012). Some detailed food items have non-standardized consumption frequency options in the FFQ and we modified them to also represent weekly consumption. For example, items “wine”, “beer”, “spirits”, and “other alcohol” have options (“> once a week”, “once a week”, “< once a week”, and “no”) that were converted to weekly frequency 2, 1,0.5, and 0 times respectively (Northstone and Emmett, 2008). Detailed algorithm descriptions can be provided upon request.

Similar to previous studies (Hu et al., 1999; Northstone and Emmett, 2005; Northstone et al., 2012; Vermeulen et al., 2016), we combined some original detailed items in the FFQ before analysis since they belong to the same food type. Thus, 87 detailed food items in the FFQ were condensed into 38 food groups (e.g. “shellfish”, “white fish without coating”, “tuna”, and “other fish” were combined into the food group “fish & other seafood”) (Supplemental Table 1). Weekly consumption frequencies for each of the 38 food groups were then used as the 38 predictors/independent variables in the RRR model.

**Inflammation.** When children were about 9.5 years old, they were invited to a clinic visit for biological assessments. Children gave blood samples after their parents provided informed consent. The blood samples were promptly spun and refrigerated at −80 ​°C. Inflammatory markers were assessed in 2008 following a storage period (median 7.5 years) without any freeze-thaw cycle (Khandaker et al., 2014). Two biomarkers of inflammation were considered in this analysis: C-reactive protein (CRP) and Interleukin 6 (IL-6). Concentration of CRP (mg/L) was assessed via the automated particle-enhanced immunoturbidimetric assay (Roche, UK) and concentration of high sensitivity IL-6 (pg/mL) was assessed via enzyme-linked immunosorbent assay (R&D systems, Abingdon, UK). For both biomarkers, inter-assay coefficients of variation were less than 5% (Khandaker et al., 2014). Among the study sample, 3814 children had valid measurements of the two inflammatory biomarkers (CRP and IL-6) at age 9.5. To improve normality, both CRP and IL-6 levels were log-transformed and then were used as the dependent variables in the RRR model (Tabung et al., 2016).

**Deriving the inflammatory dietary pattern score.** An inflammatory dietary pattern (IDP) score was derived using data on dietary intake and inflammatory biomarkers via RRR (Tabung et al., 2016). The RRR model started from the covariance matrix of the two response variables (two biomarkers) and extracted linear functions of predictors (38 food groups) that explain the shared variation in the two response variables as much as possible (Hoffmann et al., 2004). The maximum number of factors derived equals the number of response variables (Hoffmann et al., 2004) and thus two factors (or dietary patterns) were derived. The first and second factor explained 1.40% and 0.31% respectively of the shared variation in both biomarkers. In alignment with previous studies (Ambrosini et al., 2016; Hoffmann et al., 2004; Lucas et al., 2014; Tabung et al., 2016), the first factor was kept for subsequent analyses since it explains the most shared variation. Then, the food groups that are more essential to IDP were identified via stepwise linear regression with the response score of the first factor as the dependent variable (p ​< ​0.05). Finally, intakes of the several essential food groups identified via the final stepwise linear regression model were weighted by the corresponding regression coefficients in the stepwise linear regression. These weighted intakes were summed up to construct the IDP score (Lucas et al., 2014; Schulze et al., 2003; Tabung et al., 2016). The IDP score reflects the inflammatory potential of the diet, with a lower (more negative) score indicating an anti-inflammatory diet and higher (more positive) score indicating a pro-inflammatory diet. Spearman correlation coefficients were utilized to assess associations among factor score, response score, the IDP score, component food groups identified, and levels of the inflammatory biomarkers (Table 1). Among the study sample (n ​= ​6939), 3814 participants who had biomarker measures were included to derive the IDP and then an IDP score was calculated for each participant in the study sample based on his/her dietary intake. Since the range of the IDP score was narrow (−0.78, 0.34) and to also facilitate interpretation, IDP scores were converted to z-scores (mean 0, standard deviation 1) and treated as continuous predictors in regression models. The IDP score was also divided into tertiles to assess potential non-linear associations between inflammatory diet and depression risk (Miki et al., 2015).

**Table 1:** Components of IDP score and their correlations with inflammatory biomarkers among 3814 ALSPAC participants^a^.

	RRR dietary pattern response score^b^	RRR dietary pattern factor score^b^	IDP score	CRP	IL-6	Weights
RRR dietary pattern response score^b^	1.00	0.13***	0.10***	0.86***	0.82***	NA
RRR dietary pattern factor score^b^	0.13***	1.00	0.75***	0.13***	0.10***	NA
IDP score	0.10***	0.75***	1.00	0.09***	0.08***	NA
IDP score component food groups^c^						
Positive associations						
Sweetened beverages	0.04*	0.23***	0.31***	0.04*	0.03	8.22
Salad	0.02	0.17***	0.21***	0.02	0.02	31.01
Negative associations						
Root vegetables	−0.04**	−0.25***	−0.34***	−0.05**	−0.03^#^	−20.26
Nuts	−0.04**	−0.24***	−0.33***	−0.04**	−0.03*	−28.86
Whole grains	−0.05**	−0.36***	−0.48***	−0.04*	−0.05**	−9.24
Digestive biscuits	−0.05**	−0.38***	−0.48***	−0.05**	−0.03*	−29.87

IDP, Inflammatory Dietary Pattern; ALSPAC, Avon Longitudinal Study of Parents and Children; RRR, Reduced Rank Regression; CRP, C-reactive protein; IL-6, Interleukin-6; NA, Not Applicable.

^#^p ​< ​0.10; *p ​< ​0.05; **p ​< ​0.01; ***p ​< ​0.001.

a Participants with food intake information at age 8.5 years and with CRP and IL-6 measured at age 9 years were included (n ​= ​3814). Values except those in the last column are Spearman correlation coefficients. Values in the last column are 1000 times the regression coefficients for each IDP score component obtained from the last step of the stepwise linear regression analysis.

b The RRR dietary pattern was the first factor obtained from RRR with all 38 food groups. The response score is a linear function of responses (biomarkers) and the factor score is a linear function of predictors (food groups). The response score was then utilized as the dependent variable in the stepwise linear regression analyses to identify the most important food groups contributing to the IDP score.

c Six component food groups of the IDP score were identified through the stepwise linear regression with p ​< ​0.05 for inclusion and exclusion. Sweetened beverages include sweetened fruit juice, squash, cola, other fizzy drinks (e.g. lemonade, fizzy water), and flavored milk; salad includes lettuce, cucumber, peppers, other raw vegetables, etc.; root vegetables include carrots and other root vegetables (e.g. turnip, swede, parsnip); nuts include peanuts/peanut butter and other nuts (e.g. cashew, nut roast); whole grains include crispbreads, oat cereals, bran cereals, brown/granary bread, wholemeal bread, chappatis/pitta, and naan; digestive biscuits include Rich tea, shortcake, digestive and chocolate digestive, Hob Nobs, etc.

### 2.4 Covariates

Children’s race (white vs. non-white), parental highest education level based on the UK classification system (below O-level, O-level, A-level, or above A-level), and parental highest social class (professional, managerial/technical, skilled non-manual, skilled manual, or partly skilled/unskilled) were collected from maternal questionnaires issued at about 32 weeks gestation. Children’s sex (male vs. female), maternal age at delivery (<25 years, 25–29 years, 30–34 years, or ​≥ ​35 years; range: 15–44 years) (Earls and Jung, 1987), and children’s birth weight (<2500 ​g (low birthweight), 2,500 ​g-<4,000 ​g (normal birthweight), or ​≥ ​4,000 ​g (high birthweight); range: 645–5640 ​g) (World Health Organization, 2016) were collected from questionnaires and hospital records. The following confounders were assessed via maternal questionnaires (with assessment time indicated by age of children in years shown in square brackets): children’s physical activity levels (frequency the child goes to the swimming pool or other sporting area: < once a month, once a month, once a week, or at least twice a week) [5 years], maternal smoking status (smokers vs. smokers) [5 years], maternal mental health (anxiety (yes vs. no) and depression (yes vs. no)) [6 years], and maternal marital status (married vs. other) [8 years]. At 8.5 years, energy intake (EI) (kJ/day) was calculated by the ALSPAC research team from University of Bristol using the fifth edition of “McCance and Widdowson’s The composition of Food” (The Royal Society of Chemistry and Ministry of Agriculture, Fisheries and Food, 1991) based on standard age-specific portion size (Ministry of Agriculture, Fisheries and Food, 1993) and consumption of each food item collected in the FFQ (Emmett et al., 2015; Northstone et al., 2012). Among the study sample, EI ranged from 2282.82 ​kJ/day to 18866.99 ​kJ/day with mean 7945.58 ​kJ/day and standard deviation 1934.48 ​kJ/day. The total coefficient of variation (CVt) equaled 24% (Black and Cole, 2000). To better reflect biological plausibility, one CVt was utilized to specify the range of misreporting of EI based on the individualized estimated energy requirement (EER) (Ambrosini et al., 2016; Huang et al., 2005; Johnson et al., 2008). EER was calculated according to gender and age group-specific formulae with the body weight that coincided with the EI estimation (Torun, 2005). The body weight at age 8.5 years was estimated by that measured at age 9.5 years. Thus, for each child, the estimated/reported EI within 76%–124% of his/her individualized EER can be considered as plausibly reported. Participants are then divided into three groups as underreporters (EI/EER<0.76), plausible reporters (0.76 ​≤ ​EI/EER ​≤ ​1.24), or overreporters (EI/EER>1.24). At the clinic visit of age 9.5 years, height and weight were measured. BMI was calculated by dividing height in meters by square of weight in kilograms. Body weight status (underweight, normal weight, overweight, or obesity) was defined based on the age- and gender-specific BMI cut points from the International Obesity Task Force (Cole and Lobstein, 2012).

### 2.5 Statistical analysis

We conducted multiple imputation (MI) to decrease potential attrition bias while increasing statistical power. The ALSPAC includes many measures that are very useful to predict missingness of other critical variables. Thus, the assumption of “missing at random” (MAR), on which MI is based, is plausible (Bowes et al., 2015; Hammerton et al., 2015; Yuan, 2011). MI was conducted via fully conditional specification (FCS) within the statistical procedure SAS Proc MI (SAS Institute Inc.) with 10 cycles of burn-in iterations before each imputation (Bartlett et al., 2015). Suggested by previous studies (Bowes et al., 2015; White et al., 2011), the imputation model included both variables in the analysis model (exposure, outcome, and confounders) and additional variables, such as other demographic and SES factors, child mental health, child BMI, and maternal mental health indicators. The number of imputed data sets should be at least 100 times the maximum value of the fraction of missing information (FMI) that can be estimated by the maximum missing rate among the variables in the analysis model (White et al., 2011). Among the study sample, ICD-10 diagnosis of depression at early adulthood has the highest missing rate (0.50). Thus, we imputed the data set 50 times and the relative efficiency for each variable in the analysis model achieved at least 0.99.

SAS 9.4 (SAS Institute Inc., Cary, NC, USA) was utilized to conduct all statistical analyses based on the multiply imputed data sets. Associations between exposure (the IDP score in childhood) and outcome (depression in adulthood) and associations between each covariate considered in the analysis model and exposure or outcome were evaluated via bivariate analyses where the IDP score was divided into tertiles. For each categorical variable, chi-square tests were conducted over the exposure or outcome across multiply imputed data sets and the p-value was calculated based on pooled statistics from these tests (Ratitch, Lipkovich, & O’Kelly, 2013). For each continuous variable, type 3 analyses (over levels of exposure or outcome) were conducted across multiply imputed data sets and the p-value was calculated based on pooled statistics from these tests (Gantz, 2006; Wang et al., 2014). The significance level was set to be 0.10. If a covariate was significantly related to either exposure or outcome, then it was included in the adjusted statistical models.

Logistic regression models evaluated the association between the IDP score in childhood and depression in early adulthood. The IDP score was treated as a continuous z-score and also as tertiles. Trend tests were also conducted. For each form of the IDP score, four logistic models were separately constructed. Similar to previous studies (Ambrosini et al., 2016; Johnson et al., 2008; McMartin et al., 2012), model 1 was the crude model; model 2 adjusted for EI and EI misreporting status; model 3 adjusted for other covariates (sociodemographic, birth-related, maternal, child behavioral factors, and weight status); and model 4 was the fully adjusted model. Finally, like previous studies (Lucas et al., 2014; Sanchez-Villegas et al., 2015; Tabung et al., 2016), we conducted the analysis stratified by weight status (underweight and normal weight vs. overweight and obese) to investigate potential effect modification.

## 3 Results

### 3.1 IDP score

We identified six component food groups that significantly contributed to the IDP score. Spearman correlation coefficients between the IDP score and levels of inflammatory biomarkers were 0.09 (p ​< ​0.01) and 0.08 (p ​< ​0.01) for CRP and IL-6 respectively. The IDP was associated with high intakes of sweetened beverages and salad but low intakes of root vegetables, nuts, whole grains, and digestive biscuits (all p ​< ​0.01). Except for salad, all the other food groups were significantly associated with at least one inflammatory biomarker (Table 1).

### 3.2 Confounders chosen for the statistical models

As shown in Table 2, compared to participants with the lowest tertile IDP score, participants with higher IDP scores had a higher probability of developing depression in early adulthood (p ​= ​0.02; from the lowest to the highest IDP tertile, prevalence of depression was 11.7%, 12.6%, and 15.2% in each group respectively). In addition, higher proportions of participants in the higher IDP score groups were female, non-white race, less physically active, overweight or obese, and born to mothers who smoked and who had younger age at delivery (all p ​< ​0.05). Higher IDP score was also associated with lower EI derived from FFQs which tended to be underreported (p ​< ​0.05). Lower parental education level (less likely to have above A-level), lower parental social class (less likely to be professional), and non-married parents were also associated with higher IDP scores (all p ​< ​0.05) (Table 2). Depression incidence by early adulthood was higher among female participants, those with higher IDP score and BMI, those who were less physically active, and those whose mothers were smokers, had anxiety and depression and were not married (all p ​< ​0.05) (Table 3). Thus, EI misreporting status, EI, gender, race, parental education, parental social class, parental marital status, maternal age at delivery, birthweight, maternal smoking status, maternal anxiety, maternal depression, physical activity level, and weight status were included in the analysis model.

**Table 2:** Characteristics by tertiles of the IDP score among 6939 ALSPAC participants^a^.

Characteristics	Total		IDP score						P-value^b^
			1st Tertile		2nd Tertile		3rd Tertile		
	**n**	**(%)**	**n**	**(%)**	**n**	**(%)**	**n**	**(%)**	
**Depression at age 18 years**^c^									
Yes	911	(13.1)	270	(11.7)	290	(12.6)	351	(15.2)	0.020*
No	6028	(86.9)	2043	(88.3)	2023	(87.4)	1962	(84.8)	
**Socio-demographic factors**									
Sex									
Male	3488	(50.3)	1301	(56.2)	1138	(49.2)	1049	(45.4)	<.001**
Female	3451	(49.7)	1012	(43.8)	1175	(50.8)	1264	(54.6)	
Race									
White	6684	(96.3)	2246	(97.1)	2226	(96.3)	2211	(95.6)	0.039*
Non-White	255	(3.7)	67	(2.9)	87	(3.7)	102	(4.4)	
Parental education									
Below O-level	881	(12.7)	230	(9.9)	309	(13.4)	342	(14.8)	<.001**
O-level	1775	(25.6)	514	(22.2)	615	(26.6)	646	(27.9)	
A-level	2370	(34.2)	777	(33.6)	791	(34.2)	803	(34.7)	
Above A-level	1912	(27.6)	792	(34.2)	598	(25.8)	523	(22.6)	
Parental social class									
Professional	1174	(16.9)	476	(20.6)	373	(16.1)	326	(14.1)	<.001**
Managerial or technical	3163	(45.6)	1091	(47.2)	1020	(44.1)	1053	(45.5)	
Skilled non-manual	1848	(26.6)	534	(23.1)	671	(29.0)	643	(27.8)	
Skilled manual	556	(8.0)	160	(6.9)	178	(7.7)	219	(9.5)	
Partly skilled/unskilled	197	(2.8)	52	(2.3)	72	(3.1)	73	(3.2)	
Parental marital status									
Married	5613	(80.9)	1946	(84.1)	1848	(79.9)	1820	(78.7)	<.001**
Not married	1326	(19.1)	367	(15.9)	465	(20.1)	493	(21.3)	
**Birth-related factors**									
Maternal age at delivery									
<25 years	992	(14.3)	277	(12.0)	331	(14.3)	383	(16.6)	<.001**
25–29 years	2731	(39.4)	890	(38.5)	913	(39.5)	928	(40.1)	
30–34 years	2365	(34.1)	850	(36.8)	777	(33.6)	738	(31.9)	
35- years	851	(12.3)	296	(12.8)	292	(12.6)	263	(11.4)	
Birthweight									
Low birthweight (<2500 ​g)	298	(4.3)	96	(4.2)	110	(4.7)	92	(4.0)	0.083^#^
Normal birthweight (2500 ​g - ​< ​4000 ​g)	5699	(82.1)	1875	(81.1)	1924	(83.2)	1899	(82.1)	
High birthweight (≥ 4000 ​g)	942	(13.6)	341	(14.8)	279	(12.1)	321	(13.9)	
**Maternal factors**									
Smoking status									
Smoker	1407	(20.3)	406	(17.6)	494	(21.4)	506	(21.9)	<.001**
Non-smoker	5532	(79.7)	1907	(82.4)	1819	(78.6)	1807	(78.1)	
Anxiety									
Yes	1437	(20.7)	456	(19.7)	472	(20.4)	509	(22.0)	0.164
No	5502	(79.3)	1857	(80.3)	1841	(79.6)	1804	(78.0)	
Depression									
Yes	1497	(21.6)	482	(20.8)	502	(21.7)	513	(22.2)	0.543
No	5442	(78.4)	1831	(79.2)	1811	(78.3)	1800	(77.8)	
**Child behavioral factors**									
Physical activities									
Less than once a month	2109	(30.4)	655	(28.3)	695	(30.0)	759	(32.8)	0.019*
Once a month	1934	(27.9)	643	(27.8)	667	(28.8)	624	(27.0)	
Once a week	2448	(35.3)	858	(37.1)	817	(35.3)	772	(33.4)	
At least twice a week	449	(6.5)	157	(6.8)	134	(5.8)	158	(6.8)	
Energy intake									
Underreport	1248	(18.0)	250	(10.8)	435	(18.8)	562	(24.3)	<.001**
Plausible report	4479	(64.5)	1521	(65.8)	1515	(65.5)	1442	(62.4)	
Overreport	1214	(17.5)	542	(23.4)	362	(15.7)	309	(13.3)	
Energy intake (kJ/day)									
Mean (SE)	7946	(23.22)	8511	(39.54)	7803	(39.11)	7523	(39.34)	<.001**
**Child weight status**									
BMI									
Underweight	603	(8.7)	202	(8.7)	213	(9.2)	187	(8.1)	<.001**
Normal weight	4895	(70.5)	1715	(74.1)	1609	(69.5)	1571	(67.9)	
Overweight	1189	(17.1)	331	(14.3)	407	(17.6)	452	(19.5)	
Obesity	252	(3.6)	65	(2.8)	84	(3.6)	103	(4.4)	

IDP, Inflammatory Dietary Pattern; ALSPAC, Avon Longitudinal Study of Parents and Children.

^#^p ​< ​0.10; *p ​< ​0.05; **p ​< ​0.01.

a Imputed sample, n ​= ​6939 (number of imputations: 50). Participants without depressive disorders and with IDP score at baseline were included.

b For categorical characteristics, p-values were obtained based on chi-squared tests pooled across multiply imputed samples. For continuous characteristics (e.g. birthweight), p-values were obtained based on type 3 analyses pooled across multiply imputed samples.

c Depression cases were identified if participants had ICD-10 diagnosis or had depression score ​≥ ​9 (out of 21) in the Computerized Interview Schedule-Revised (CIS-R) at early adulthood.

**Table 3:** Characteristics by depression status among 6939 ALSPAC participants^a^.

Characteristics	Depression at age 18 years^b^		P-value^c^
	n	(%)	
IDP score			
1st Tertile	270	(11.7)	0.020*
2nd Tertile	290	(12.6)	
3rd Tertile	351	(15.2)	
**Socio-demographic factors**			
Sex			
Male	348	(10.0)	<.001**
Female	563	(16.3)	
Race			
White	875	(13.1)	0.527
Non-White	36	(14.2)	
Parental education			
Below O-level	116	(13.2)	0.553
O-level	234	(13.2)	
A-level	328	(13.9)	
Above A-level	232	(12.1)	
Parental social class			
Professional	129	(11.0)	0.227
Managerial or technical	428	(13.5)	
Skilled non-manual	242	(13.1)	
Skilled manual	86	(15.5)	
Partly skilled/unskilled	26	(13.1)	
Parental marital status			
Married	682	(12.1)	0.001**
Not married	229	(17.3)	
**Birth-related factors**			
Maternal age at delivery			
<25 years	151	(15.2)	0.417
25–29 years	356	(13.0)	
30–34 years	294	(12.4)	
35- years	110	(13.0)	
Birthweight			
Low birthweight (<2500 ​g)	32	(10.8)	0.493
Normal birthweight (2500 ​g - ​< ​4000 ​g)	759	(13.3)	
High birthweight (≥ 4000 ​g)	120	(12.7)	
**Maternal factors**			
Smoking status			
Smoker	236	(16.8)	0.003**
Non-smoker	675	(12.2)	
Anxiety			
Yes	246	(17.1)	<.001**
No	665	(12.1)	
Depression			
Yes	240	(16.0)	0.009**
No	671	(12.3)	
**Child behavioral factors**			
Physical activities			
Less than once a month	317	(15.0)	0.044*
Once a month	265	(13.7)	
Once a week	282	(11.5)	
At least twice a week	48	(10.6)	
Energy intake			
Underreport	175	(14.0)	0.616
Plausible report	582	(13.0)	
Overreport	155	(12.7)	
Energy intake (kJ/day)			
Mean (SE)	7872	(88.3)	0.103
**Child weight status**			
BMI			
Underweight	75	(12.5)	0.265
Normal weight	615	(12.6)	
Overweight	178	(14.9)	
Obesity	43	(17.1)	

ALSPAC, Avon Longitudinal Study of Parents and Children; IDP, Inflammatory Dietary Pattern.

^#^p ​< ​0.10; *p ​< ​0.05; **p ​< ​0.01.

a Imputed sample, n ​= ​6939 (number of imputations: 50). Participants without depressive disorders and with IDP score at baseline were included.

b Depression cases were identified if participants had ICD-10 diagnosis or had depression score ​≥ ​9 (out of 21) in the Computerised Interview Schedule-Revised (CIS-R) at early adulthood.

c For categorical characteristics, p-values were obtained based on chi-squared tests pooled across multiply imputed samples. For continuous characteristics (e.g. birthweight), p-values were obtained based on type 3 analyses pooled across multiply imputed samples.

### 3.3 Associations between IDP score in childhood and depression at early adulthood

Among 6939 children who were free from depression at baseline, 911 (13.1%) incident depression cases were identified in their early adulthood after a 10-year follow-up. One-unit increase in the IDP z-score corresponded to a 10% increase in the odds of developing depression (OR ​= ​1.10; 95% CI: 1.01–1.21). After EI misreporting status and EI was adjusted, the association was nearly unchanged (OR ​= ​1.10; 95% CI: 1.00–1.21). The relationship was attenuated after socio-demographics, birth-related factors, maternal factors, child behavioral factors, and child weight status were adjusted. When children were classified based on tertiles of the IDP score, participants in the highest tertile had 1.35 times the odds of developing depression (95% CI: 1.10–1.67) compared to those in the lowest tertile. The association remained relatively unchanged after dietary misreporting status and EI were adjusted (OR ​= ​1.34; 95% CI: 1.08–1.66). A dose-response relation between IDP tertiles and depression risk was also observed (p-trend ​= ​0.008). However, the association between IDP tertiles and depression became marginally significant as covariates were added to the model (OR ​= ​1.22; 95% CI: 0.98–1.52 [highest tertile vs. lowest tertile]; p-trend ​= ​0.088) and only the test for trend was marginally significant in the fully adjusted model (p-trend ​= ​0.097) (Table 4).

**Table 4:** Associations between IDP score in childhood and depression in early adulthood among 6939 ALSPAC participants^a^.

	Model 1^b^		Model 2^c^		Model 3^d^		Model 4^e^	
	OR	95% CI	OR	95% CI	OR	95% CI	OR	95% CI
IDP z-score (continuous)	1.10	(1.01, 1.21)	1.10	(1.00, 1.21)	1.05	(0.95, 1.16)	1.04	(0.95, 1.15)
IDP score tertiles								
1st Tertile	1.00		1.00		1.00		1.00	
2nd Tertile	1.09	(0.88, 1.35)	1.08	(0.87, 1.34)	1.02	(0.82, 1.27)	1.01	(0.81, 1.26)
3rd Tertile	1.35	(1.10, 1.67)	1.34	(1.08, 1.66)	1.22	(0.98, 1.52)	1.21	(0.96, 1.51)
P for trend	0.005		0.008		0.088		0.097	

IDP, Inflammatory Dietary Pattern; ALSPAC, Avon Longitudinal Study of Parents and Children; OR, Odds Ratio; CI, Confidence Interval.

a Imputed sample, n ​= ​6939 (number of imputations: 50). Participants without depressive disorders and with IDP score at baseline were included. Depression cases were identified if participants had ICD-10 diagnosis or had depression score ​≥ ​9 (out of 21) in the Computerized Interview Schedule-Revised (CIS-R) at early adulthood.

b Crude model.

c Adjusted for energy intake misreporting (underreporter, plausible reporter, or overreporter) and energy intake (range: 2282.82–18866.99 ​kJ/d).

d Adjusted for sex (female vs. male), race (non-white vs white), parental education (below O-level, O-level, A-level, or above A-level), parental social class (professional, managerial/technical, skilled non-manual, skilled manual, or partly skilled/unskilled), parental marital status (married vs. other), maternal age at delivery (range: 15–44 years), birthweight (range: 645–5640 ​g), maternal smoking status (yes vs. no), maternal anxiety (yes vs. no), maternal depression (yes vs. no), physical activities level (frequency the child goes to the swimming pool or other sporting area: < once a month, once a month, once a week, or at least twice a week), and weight status (underweight, normal weight, overweight, or obesity).

e Fully adjusted model (i.e. Model 3 plus energy intake misreporting (underreporter, plausible reporter, or overreporter) and energy intake (range: 2282.82–18866.99 ​kJ/d)).

The relationships between IDP score (the continuous IDP z-score and the IDP tertiles) and incident depression were stronger among participants with under or normal weight compared with those associations among participants who were overweight or obese. For example, among participants with under or normal weight, those in the highest tertile had 1.27 times the odds of developing depression in early adulthood (95% CI: 0.98–1.65) compared to those in the lowest tertile after all covariates were adjusted, while the corresponding OR was 0.99 (95% CI: 0.61–1.60) among participants who were overweight or obese. In addition, no significant associations between IDP score and incident depression were observed among participants with overweight and obesity (Table 5).

**Table 5:** Associations between IDP score in childhood and depression in early adulthood among 6939 ALSPAC participants stratified by weight status^a^.

Weight status	Model 1^b^		Model 2^c^		Model 3^d^		Model 4^e^	
	OR	95% CI	OR	95% CI	OR	95% CI	OR	95% CI
Underweight or normal weight (n ​= ​5498)								
IDP z-score (continuous)	1.13	(1.02, 1.25)	1.12	(1.01, 1.24)	1.08	(0.97, 1.20)	1.07	(0.96, 1.20)
IDP score tertiles								
1st Tertile	1.00		1.00		1.00		1.00	
2nd Tertile	1.20	(0.93, 1.55)	1.18	(0.92, 1.53)	1.14	(0.87, 1.47)	1.13	(0.87, 1.46)
3rd Tertile	1.40	(1.10, 1.80)	1.38	(1.08, 1.78)	1.29	(0.99, 1.67)	1.27	(0.98, 1.65)
P for trend	0.008		0.011		0.056		0.070	
Overweight or obese (n ​= ​1441)								
IDP z-score (continuous)	0.99	(0.82, 1.20)	0.98	(0.80, 1.21)	0.95	(0.77, 1.16)	0.94	(0.76, 1.16)
IDP score tertiles								
1st Tertile	1.00		1.00		1.00		1.00	
2nd Tertile	0.74	(0.47, 1.17)	0.73	(0.45, 1.16)	0.69	(0.43, 1.10)	0.68	(0.42, 1.10)
3rd Tertile	1.10	(0.71, 1.70)	1.08	(0.68, 1.71)	1.00	(0.63, 1.60)	0.99	(0.61, 1.60)
P for trend	0.549		0.595		0.836		0.872	

IDP, Inflammatory Dietary Pattern; ALSPAC, Avon Longitudinal Study of Parents and Children; OR, Odds Ratio; CI, Confidence Interval.

a Imputed sample, n ​= ​6939 (number of imputations: 50). Participants without depressive disorders and with IDP score at baseline were included. Depression cases were identified if participants had ICD-10 diagnosis or had depression score ​≥ ​9 (out of 21) in the Computerized Interview Schedule-Revised (CIS-R) at early adulthood.

b Crude model.

c Adjusted for energy intake misreporting (underreporter, plausible reporter, or overreporter) and energy intake (range: 2282.82–18866.99 ​kJ/d).

d Adjusted for sex (female vs. male), race (non-white vs white), parental education (below O-level, O-level, A-level, or above A-level), parental social class (professional, managerial/technical, skilled non-manual, skilled manual, or partly skilled/unskilled), parental marital status (married vs. other), maternal age at delivery (range: 15–44 years), birthweight (range: 645–5640 ​g), maternal smoking status (yes vs. no), maternal anxiety (yes vs. no), maternal depression (yes vs. no), and physical activities level (frequency the child goes to the swimming pool or other sporting area: < once a month, once a month, once a week, or at least twice a week).

e Fully adjusted model (i.e. Model 3 plus energy intake misreporting (underreporter, plausible reporter, or overreporter) and energy intake (range: 2282.82–18866.99 ​kJ/d)).

## 4 Discussion

In this large prospective cohort study, we synthesized biomarkers of inflammation with dietary behavioral information measured in childhood to empirically derive an inflammatory dietary pattern score for children who were free from depression at baseline. We found that after a 10-year follow-up period, depression risk increased as the inflammatory diet score increased, indicating that children with diets characterized by higher intake of pro-inflammatory foods may be at heightened risk of developing depression later in life. However, it is important to note that some of the associations were attenuated with full covariate adjustment. Thus, this study provides some preliminary evidence that adulthood depression risk may have some origins in inflammatory dietary patterns established in childhood. We encourage future work to build on this emerging evidence and continue to consider associations between early life inflammatory diets and the emergence of depression across the life course.

Based on previous studies (Ambrosini et al., 2016; Lucas et al., 2014; Northstone and Emmett, 2008), we expected that unhealthy food groups, such as fizzy drinks, white bread, cakes, and red meat, would be positively related to IDP score while healthy food groups, such as vegetables, nuts, and wholegrain bread, would be negatively related to IDP score. The six food groups identified as critical components of the IDP generally aligned with the expectation, except for salad which was positively related to IDP score. This counterintuitive finding could be due to the consumption of salad dressings, which often contain oils high in omega-6 fatty acids (i.e., soybean, corn, and safflower oils) and could be pro-inflammatory (DiNicolantonio & O’Keefe, 2018; Innes and Calder, 2018; Marchix et al., 2015; Patterson et al., 2012). Previous studies have identified other vegetables (e.g., corn, celery, mushrooms, green pepper, eggplant, summer squash, and mixed vegetables) as positively contributing to the IDP score (Lucas et al., 2014; Tabung et al., 2016). Further investigation is warranted to unravel the exact contents within the “salad” grouping. Method of food preparation should also be considered. In addition, based on previous studies that suggest food items be combined into food groups (Lucas et al., 2014; Northstone and Emmett, 2005; Northstone and Emmett, 2008; Tabung et al., 2016), we classified 90 food items into 38 food groups. Although the classification was subjective and can be done in multiple ways, it was verified by a registered dietitian co-investigator. Different methods for food group classification can be conducted in future research to further detect sensitivity of the current statistical method to derive IDP.

We found that only a small proportion of variation among levels of inflammatory biomarkers (2%) was explained by the dietary patterns extracted via RRR. Though low, this level is similar to what has been observed in prior work. For example, a previous study indicated that among middle aged and older women enrolled in NHS, only 4% of variation among inflammatory biomarkers was explained by empirically derived dietary patterns (Tabung et al., 2016). Compared with middle aged and older adults, children tend to be healthier and have lower levels of inflammation, which can help explain why a smaller amount of variation among inflammatory biomarkers was explained by dietary intake in this sample. Additionally, IDP in the current study was constructed based on the only two inflammatory biomarkers (CRP and IL-6) available in the ALSPAC data set. In the NHS-based study, researchers also included tumor necrosis factor α receptor 2 (TNFαR2) in addition to CRP and IL-6 as inflammatory biomarkers and thus constructed the RRR model based on three response variables to derive IDP (Tabung et al., 2016), which may contribute to the difference between the two studies in the proportion of variation among biomarkers explained by IDP. In future studies, we encourage researchers to incorporate a broad panel of inflammatory markers in deriving IDP scores in order to enhance the sensitivity of the measure.

Contrary to our expectations of finding a stronger effect of inflammatory diet on depression among those who were overweight or obese, our findings suggest the opposite. One possible explanation for this counterintuitive finding could be that compared to children with overweight or obesity, children with under or normal weight may have more variability in inflammation in response to their dietary intake and thus the effect of IDP on depression risk may be more easily detected. Adipose tissue produces inflammatory factors, including cytokines such as IL-6, TNF-α, and chemokines, which in turn may activate widespread immune reaction, potentially leading to or exacerbating inflammation-related diseases including depression (Shelton and Miller, 2011). Obesity is thus a pro-inflammatory condition and can cause a chronic low-grade inflammation state (Hotamisligil, 2006; Ouchi et al., 2011), which could potentially obscure any relationship between diet-induced inflammation and depression risk. An alternative possible explanation for the null findings among the overweight/obese group could be the statistical power. The 95% CIs among the overweight/obese group were much wider than those in the under/normal weight group since the sample size for overweight or obese children is relatively small (about 20% of the study sample) compared to that for children with under or normal weight, which may lead to lower power to detect the effect. Thus, the different associations observed across weight strata in this study could be attributable to existing levels of inflammation and/or power considerations. We encourage future research to consider these alternatives explicitly.

The IDP score allows for an integration of behavioral and physiologic factors that can jointly explain the incidence of depression. Intake of inflammation-inducing foods may lead to higher serum levels of inflammatory cytokines, such as IL-6, which have been shown to boost activation of the hypothalamic-pituitary-adrenal (HPA) axis and to increase oxidative stress in the brain. These effects could be responsible for the impaired mood and feelings observed in depression cases (Dantzer, O’Connor, Freund, Johnson and Kelley, 2008; Miller et al., 2009). Future work with measured markers of HPA-related activity should explicitly test the possibility that the IDP may trigger dysregulation of the HPA axis.

The current study has some significant advantages. First, it is a population-based study with large sample size. Second, it prospectively investigates and establishes the temporal relationship between IDP in childhood and depression risk in early adulthood. Third, participants were followed from age 9 to age 18, with covariate information measured from birth. Due to this lengthy follow-up time, long-term effects from IDP in childhood on mental health in young adulthood can be examined over the life course. Fourth, various familial and socio-demographic factors are adjusted for and the independent influence of childhood IDP on depression in youth can be examined. In addition, using RRR took advantage of objectively measured biomarkers to derive an inflammation prone dietary pattern, demonstrating the role of chronic inflammation in the etiology of depression through diet in early life.

However, the study also has several limitations. First, loss-to-follow-up is a concern for prospective studies. Young adults who came from families with higher SES and had better childhood health were more likely to attend clinic visits to have measurements for IL-6 or CRP and depressive symptoms, which raises concerns for selection effects. Second, only food items provided in home were included in the analysis and foods provided by others (e.g. at school) were not included, which may affect the precision of the dietary measure used in this study. Third, methods to prepare food were not assessed in the FFQ; it is unknown whether healthy food items were prepared in an unhealthy way. In addition, food intake was based on maternal report, and measurement errors cannot be ruled out. Dietary intake measured at a single time point was used, which may not represent dietary practices across childhood. However, prior work in the cohort indicates a high correlation of dietary scores across childhood which somewhat mitigates this concern (Northstone and Emmett, 2008). Finally, generalizability of the study is limited because about 95% of participants are White.

The physical health benefits of healthy eating are well known and are the focus of much public health research, practice, and intervention (Rodríguez-Monforte et al., 2015; Rosato et al., 2019; USDA & HHS, 2016). The results of this study suggest that healthy dietary practices, particularly when established early in life, may provide a mental health benefit as well. The current study illustrates the potential developmental origins of depression from the joint perspective of inflammation and diet. Study findings provide preliminary evidence that pro-inflammatory diets in early life may increase depression risk, which in turn suggests that interventions designed to promote anti-inflammatory diets could provide mental health benefits to children that persist into adulthood. Future research should build on these results and work to provide new insights into the role of diet in relation to depression prevention and mental health promotion across the life course.

## Funding/Support

The UK Medical Research Council and the Wellcome Trust (Grant ref: 102215/2/13/2) and the University of Bristol provide core support for ALSPAC. The Wellcome Trust (Grant ref: 08426812/Z/07/Z) funds CIS-R assessment. This research was partially supported by a grant to the Center for Social and Demographic Analysis (CSDA) at the University at Albany from the 10.13039/100000071National Institute of Child Health and Human Development (R24-HD044943).

## Role of the sponsors

The funding sources were not involved in the data analysis, manuscript writing, and publication. This publication is the work of the authors, who will serve as guarantors for the contents of this paper.

## Declaration of competing interest

None reported.

## Supplementary data

XC_20191109_IDP and dep_supplemental materials XC_20191109_IDP and dep_supplemental materials
