# Supplementary material for: The relationship between inflammatory dietary pattern in childhood and depression in early adulthood
Source: Brain Behav Immun Health. 2019 Dec 15;2:100017. doi: 10.1016/j.bbih.2019.100017 (PMC7043331; doi:10.1016/j.bbih.2019.100017)
Supplement: XC_20191109_IDP and dep_supplemental materials [file mmc1.docx]

Author: Xiao Cong; Title: Inflammatory Dietary Pattern and Depression

**Appendix A. Supplementary Data**

| **Supplemental Table 1.** Food groups (n=38) and their component food items (n=87). | | | |
| --- | --- | --- | --- |
| Food categories | Food groups | Food items | Number of food items in each food group |
| Meat & Seafood | Processed meats | sausages/burgers, meat pies or pasties, ham/bacon/cold meats, chicken or turkey in crispy coating, white fish in breadcrumbs/batter | 5 |
|  | Meat dishes | meat roasts/chops/stews | 1 |
|  | Offals | liver/liver pate, kidney/heart | 2 |
|  | Poultry | poultry | 1 |
|  | Fish & other seafood | shellfish, white fish without coating, tuna, other fish | 4 |
| Dairy | Eggs | eggs/quiche/omelettes/flan | 1 |
|  | Cheese | cheese | 1 |
|  | High-fat dairy products | full fat milk | 1 |
|  | Low-fat dairy products | semi-skimmed milk, skimmed milk, yoghurt | 3 |
| Produce | Legumes | baked beans, peas or broad beans, pulses, soya meat | 4 |
|  | Sweetcorn | sweetcorn | 1 |
|  | Green vegetables | cabbage/Brussel sprouts/spinach, other green vegetables | 2 |
|  | Root vegetables | carrots, other root vegetables | 2 |
|  | Tomatoes | tomatoes | 1 |
|  | Salad | salad (lettuce, cucumber, peppers, other raw vegetables, etc.) | 1 |
|  | Nuts | peanuts or peanut butter, other nuts | 2 |
|  | Fruits | fresh citrus fruit, other fresh fruit, canned fruit | 3 |
|  | Fruit juice | pure fruit juice | 1 |
| Staple food | Vegetarian pies | vegetarian pies or pasties | 1 |
|  | Pizza | pizza | 1 |
|  | Potatoes | oven chips, roast potatoes, boiled/mashed/jacket potatoes | 3 |
|  | Fried food | fried chips/potato waffles/croquettes, fried food | 2 |
|  | Rice | rice | 1 |
|  | Canned pasta | canned pasta | 1 |
|  | Pasta | boiled pasta | 1 |
|  | Whole grains | crispbreads, oat cereals, bran cereals, brown/granary bread, wholemeal bread, chappatis/pitta, naan | 7 |
|  | Refined grains | other cereals, white bread, soft grain white bread | 3 |
| Snacks | Sweets and desserts | milk puddings, ice cream, ice lollies, pudding, custard/cream/tip-top, cakes/buns, full-coated chocolate biscuits, chocolate bars/buttons, sweets | 9 |
|  | Crisps | crisps or corn snacks | 1 |
|  | Digestive biscuits | other biscuits (Rich tea, shortcake, digestive and chocolate digestive, Hob Nobs, etc.) | 1 |
| Drinks | Sweetened beverages | sweetened fruit juice, squash, cola, other fizzy drinks, flavored milk | 5 |
|  | Water | plain water | 1 |
|  | Tea and Coffee | tea, coffee | 2 |
|  | Herbal tea | herbal tea | 1 |
|  | Alcohol | wine, beer, spirits, other alcohol | 4 |
| Spread | Butter | butter/ghee/dripping/lard | 1 |
|  | Margarine | polyunsaturated margarine, hard or soft margarine, low-fat spread | 3 |
|  | Vegetable oil | sunflower/corn/soya oil, olive/hazelnut/rapeseed oil, other vegetable oil | 3 |
| Total |  |  | 87 |
